# Plasma concentrations of granulocyte colony-stimulating factor (G-CSF) in patients with substance use disorders and comorbid major depressive disorder

**DOI:** 10.1038/s41598-021-93075-1

**Published:** 2021-07-01

**Authors:** Sandra Torres Galván, María Flores-López, Pablo Romero-Sanchiz, Nerea Requena-Ocaña, Oscar Porras-Perales, Raquel Nogueira-Arjona, Fermín Mayoral, Pedro Araos, Antonia Serrano, Roberto Muga, Francisco Javier Pavón, Nuria García-Marchena, Fernando Rodríguez de Fonseca

**Affiliations:** 1 Laboratorio de Medicina Regenerativa, Unidad de Gestión Clínica de Salud Mental, Hospital Regional Universitario de Málaga, Avda. Carlos Haya 82, sótano, 29010 Málaga, Spain; 2 Instituto de Investigación Biomédica de Málaga-IBIMA, Málaga, Spain; 3 Facultad de Farmacia, Universidad Complutense de Madrid, Madrid, Spain; 4 Facultad de Psicología, Universidad de Málaga, Málaga, Spain; 5 Department of Psychology, University of Roehampton, London, UK; 6 Unidad de Gestión Clínica del Corazón, Hospital Universitario Virgen de La Victoria, Málaga, Spain; 7 Unidad de Adicciones- Servicio de Medicina Interna. Institut D’Investigació en Ciències de La Salut Germans Trias I Pujol (IGTP), Campus Can Ruti, Carrer del Canyet s/n, 08916 Badalona, Spain; 8 Departamento de Medicina, Universitat Autònoma de Barcelona, Barcelona, Spain; 9 Centro de Investigación Biomédica en Red Enfermedades Cardiovasculares (CIBERCV), Instituto de Salud Carlos III, Madrid, Spain

**Keywords:** Molecular biology, Biomarkers

## Abstract

Granulocyte colony–stimulating factor (G-CSF) has raised much interest because of its role in cocaine addiction in preclinical models. We explored the plasma concentrations of G-CSF in patients diagnosed with substance use disorder (SUD) and highly comorbid psychiatric disorders. In particular, we investigated the association between G-CSF concentrations and comorbid major depressive disorder (MDD) in patients with cocaine and alcohol use disorders (CUD and AUD, respectively). Additionally, patients with MDD but not SUD were included in the study. Three hundred and eleven participants were enrolled in this exploratory study: 136 control subjects, 125 patients with SUD (SUD group) from outpatient treatment programs for cocaine (N = 60, cocaine subgroup) and alcohol (N = 65, alcohol subgroup), and 50 patients with MDD but not SUD (MDD group) from primary-care settings. Participants were assessed based on DSM-IV-TR criteria, and a blood sample was collected to examine the plasma concentrations of G-CSF. G-CSF concentrations were negatively correlated with age in the entire sample (r = − 0.233, *p* < 0.001) but not in the patients with MDD. G-CSF concentrations were lower in patients with SUD than in controls (*p* < 0.05), specifically in the cocaine subgroup (*p* < 0.05). Patients with SUD and comorbid MDD had lower G-CSF concentrations than patients with SUD but not comorbid MDD or controls (*p* < 0.05). In contrast, patients with MDD but not SUD showed no differences compared with their controls. The negative association between G-CSF concentrations and age in the sample was not observed in patients with MDD. G-CSF concentrations were decreased in patients with SUD and comorbid MDD but not in patients with MDD. Therefore, G-CSF may be useful to improve the stratification of patients with dual diagnosis seeking treatment. Further investigation is needed to explore the impact of sex and type of drug on the expression of G-CSF.

## Introduction

Substance use disorder (SUD) is a chronic neurobiological-based medical illness contributing to almost 6% of all deaths worldwide^1^. In recent years, it has been confirmed that SUDs are associated with neuroinflammation, a process that can cause long-term behavioral alterations by activating glial cells, mainly microglia and astrocytes, and modifying the plasticity of the central nervous system (CNS)^2,3^. Alcohol and cocaine are two of the most commonly used drugs, both of which are well known to cause neuroinflammation; in fact, several studies have explored how these inflammatory effects contribute to the clinical course of SUDs and comorbid pathologies^3–6^.

Regarding alcohol, its pharmacological actions in the CNS include multiple neurotoxic effects, such as activation of microglial cells, alterations in the release of mediators [e.g., brain-derived neurotrophic factor (BDNF)]^4^, modifications of the lipid membrane causing alteration of the cell membrane permeability, changes in proliferation and maturation of neurons, and eventually neuronal apoptosis^5–7^. Interestingly, alcohol directly induces the activation of proinflammatory immune signaling, as revealed by the characterization of Toll-like receptor 4 (TLR4)-dependent glial activation responses associated with alcohol exposure^8^. This finding supported the inflammatory view of alcohol-induced toxic effects: preclinical studies based on reward and alcohol withdrawal behavioral responses have revealed that there are several proinflammatory mediators involved in the regulation of alcohol effects^8–11^. For example, the chemokine monocyte chemoattractant protein-1 (MCP-1) secreted by glial cells was found to be associated with alcohol consumption and alcohol-related neurodegeneration^12,13^. Other studies monitoring plasma concentrations of immune mediators have shown alcohol-induced dysregulation of certain chemokines related to immune responses such as SDF-1 [stromal derived factor (CXCL12)] and fractalkine (CX_3_CL1), which were found to be decreased in patients with alcohol use disorder (AUD)^14^. Finally, important neurotrophic factors such as BDNF and insulin-like growth factor-1 (IGF-1) were found to be profoundly affected in abstinent patients with AUD^4,15^.

In contrast, cocaine is a stimulant substance in the CNS with a high potential for abuse^16^. Cocaine consumption is associated with neuronal cytotoxicity and altered neurotrophic responses through a nitric oxide-mediated signaling pathway, among other mechanisms^17^. However, although cocaine use disorder (CUD) is mostly characterized by dysfunctions in reward-related brain circuits and dysregulation of dopaminergic reward pathways and glutamate receptor-dependent signaling cascades^18,19^, multiple studies have also described immune signaling alterations associated with cocaine use^8^. Regarding circulating immunological mediators related to cocaine consumption, proinflammatory cytokines such as tumor necrosis factor-alpha (TNF-α) were found to be elevated in plasma while the anti-inflammatory interleukin 10 (IL-10) was found to be decreased and linked to cocaine-related chronic stress^20^. Moreover, plasma concentrations of interleukin 1β (IL1β), fractalkine and SDF-1 were found to predict the severity of CUD symptoms, which suggests a relevant contribution of the immune system to cocaine addiction^21^.

The contribution of the immune system in addiction not only has been associated with the severity of SUD or the toxic effects of substances but also it might contribute to the development of comorbid disorders. Patients with SUD are more likely to suffer comorbid psychiatric disorders throughout their lives than the general population^22,23^, which affects the course of each psychiatric condition^24^. Mood, anxiety, psychotic, and personality disorders are the most prevalent diagnoses found in patients with SUD^25–27^. The dysregulation of inflammatory and immune signaling has been found to be associated with several psychiatric disorders, mainly mood disorders such as major depressive disorder (MDD)^28,29^. Immunological and affective regulatory interactions are an important aspect of MDD development^30^. Previously, it has been reported that the gene expression of IL-17, IL-21, IL-23, and IL-35 was substantially higher in patients with MDD than in controls, which suggests that the expression of specific inflammatory genes may be a factor in the etiopathogenesis of depressive disorders. Additionally, these cytokines may affect the metabolism of neurotransmitters and neuroendocrine functions in the brain and may be markers and new potential therapeutic targets for recurrent depressive disorders^31,32^.

The search for immune mediators participating in the natural history of addiction has brought attention to new molecules such as the granulocyte colony-stimulating factor (G-CSF), a glycoprotein that acts as a growth factor, cytokine and hormone in the bone marrow, blood and CNS^33^. G-CSF participates in the proliferation and differentiation of myeloid progenitors^33^, promotes the generation of granulocyte precursors and exerts antiapoptotic actions^34,35^. In the CNS, G-CSF receptors have been studied because of their neuroprotective properties^36^, including antiinflammatory effects on dopaminergic neurons in neurodegenerative disorders such as Parkinson’s disease^37^ and Alzheimer’s disease^38^. Unlike other inflammatory signals, few studies examining G-CSF have been reported in the context of mood disorders, and the only study in patients suffering MDD with long-term symptomatology found no differences in serum G-CSF relative to controls^39^. Regarding addiction, G-CSF was found to be a relevant signal that promoted cocaine self-administration and consolidates cocaine seeking behavior^40^. However, alterations in this immunomodulatory growth factor in patients with SUD have been inconclusive, evidenced by a recent meta-analysis of 74 studies has recently reported^41^.

The impact of addictive disorders in the health system emphasizes the importance of effective prevention programs and treatments^42^. Because several studies have reported a systemic inflammatory state in patients with SUD and common psychiatric disorders such as MDD, we explored whether circulating concentrations of G-CSF in the plasma were altered in patients with SUD (CUD and AUD) and psychiatric comorbidity from outpatient treatment programs, as well as in patients with MDD but not SUD from primary-care settings in Spain.

## Materials and methods

### Participants and recruitment

Three hundred thirty-five volunteers were originally enrolled in this exploratory study: 140 healthy control subjects, 140 abstinent patients diagnosed with SUD and 55 patients diagnosed with MDD. However, 24 participants were excluded because they did not meet the participation criteria (4 controls, 15 patients with SUD and 5 patients with MDD).

Control subjects (N = 136) were recruited from two different sources, a multidisciplinary staff cohort of volunteers working at the Spanish National Public Health System (i.e., *Hospital Regional Universitario de Málaga*, Málaga, Spain) and a second cohort obtained from volunteers donating data and plasma to the National Biobank of DNA (*Banco Nacional de ADN Carlos III*, Salamanca, Spain). Control subjects were divided into two groups based on the age, sex, and body mass index (BMI) of patients with SUD (N = 92, control group for SUD) and MDD (N = 44, control group for MDD). Notably, the control group for SUD was sex-balanced and showed no statistically significant differences in age and BMI with the SUD group, and the control group for MDD showed no statistically significant differences in sex, age and BMI with the MDD group.

Patients with SUD (N = 125, SUD group) were recruited from outpatient treatment programs for alcohol (N = 65, alcohol subgroup with AUD) and cocaine (N = 60, cocaine subgroup with CUD), and patients with MDD (N = 50, MDD group) were recruited from primary-care settings at the Spanish National Public Health System (*Hospital Regional Universitario de Málaga*, Málaga, Spain; *Centro Provincial de Drogodependencias*, Málaga, Spain; and *Hospital Universitario 12 de Octubre*, Madrid, Spain).

Participation in the study was voluntary, and the participants had to meet eligibility based on the following inclusion criteria: 18 years of age or older (up to 65 years) for all patients, diagnosis of AUD or CUD (a minimum of 4 weeks of abstinence) for the SUD group and diagnosis of MDD (last two months of depressive symptoms) for the MDD group. Lifetime SUD and MDD were diagnosed based on the DSM-IV-TR criteria. The exclusion criteria included personal history of chronic inflammatory diseases or cancer, infectious diseases, severe mental disorders precluding evaluation, pregnancy or breastfeeding and less than 4 weeks of abstinence from any drug, except for nicotine and caffeine. Psychiatric comorbidity was accepted for the SUD and MDD groups (i.e., additional SUD and mental disease, respectively). Regarding healthy control subjects, the exclusion criteria were identical to those for the groups of patients but also included the use of psychotropic medication during the last year and the diagnosis of psychiatric disorders based on the DSM-IV-TR criteria.

### Ethics statements

Written informed consents were obtained from each participant after a complete description of the study. All the participants had the opportunity to discuss any questions or issues. The study and protocols for recruitment were approved by the Ethics Committee of the *Hospital Regional Universitario de Malaga* in accordance with the Ethical Principles for Medical Research Involving Human Subjects adopted in the Declaration of Helsinki by the World Medical Association (64th WMA General Assembly, Fortaleza, Brazil, October 2013) and Recommendation No. R (97) 5 of the Committee of Ministers to Member States on the Protection of Medical Data (1997), and Spanish data protection act [Regulation (EU) 2016/679 of the European Parliament and of the Council 27 April 2016 on the protection of natural persons with regard to the processing of personal data and on the free movement of such data, and repealing Directive 95/46/EC (General Data Protection Regulation). All data were given code numbers in order to maintain privacy and confidentiality.

### Clinical assessments

All participants in the study were clinically evaluated by trained and experienced psychologists. The total sample was assessed using different psychiatric/clinical interviews based on the characteristics of each group, but the Spanish version of the Psychiatric Research Interview for Substance and Mental Disorders (PRISM) was typically used for all participants. The PRISM is a semistructured interview based on the DSM-IV-TR criteria with good psychometric properties in the evaluation of SUD and common comorbid psychiatric disorders in addicted patients^43,44^.

Therefore, the SUD group was specifically assessed with PRISM. The MDD group was assessed with the Beck Depression Inventory-II (BDI-II) to determine the severity of the MDD^45^ but was also assessed with PRISM to dismiss the diagnosis of SUD. Moreover, the PRISM has demonstrated good to excellent validity and test–retest reliability for differentiating substance-induced MDD (i.e., MDD as a physiological consequence of the use of substances that may appear during active use, intoxication, or withdrawal) from primary MDD, with kappa ranging from 0.66 to 0.75^43,44,44,46^. Finally, the control group was assessed with the Spanish version of the Composite International Diagnostic Interview (CIDI) for the detection of psychiatric disorders^47^ and PRISM (module 1: *Overview* for sociodemographic and physiological variables).

### Collection of plasma samples

Blood extractions were conducted under the same conditions by experienced nurses in the morning after fasting for 8–12 h. Venous blood samples were extracted into 10-mL K_2_ EDTA tubes (BD, Franklin Lakes, NJ, USA), and to obtain plasma, samples were centrifuged at 2200×*g* for 15 min (4 °C). Plasma was individually assayed by three rapid tests for detecting infectious diseases: HIV (Retroscreen HIV, QualPro Diagnostics-Tulip Group Ltd, Goa, India), hepatitis B (HBsAg Test, Toyo Diagnostics-Turklab Inc., Izmir, Turkey) and hepatitis C (Flaviscreen HCV, QualPro Diagnostics-Tulip Group Ltd). Infected samples were discarded following laboratory safety protocols. Each plasma sample was registered and stored at − 80 °C until determination of G-CSF.

### Determinations of G-CSF

Plasma concentrations of G-CSF were determined using a selective enzyme-linked immunosorbent assay (ELISA) according to the manufacturer’s instructions: Human G-CSF (Granulocyte Colony Stimulating Factor 3) ELISA kit (#EH0149, Wuhan Fine Biotech Co., Ltd. Wuhan, China). The Human G-CSF ELISA kit indicated a sensitivity < 23.4 pg/mL and a range of 39.1–2500.0 pg/mL. The experimental detection range under the experimental conditions with eight ELISA determinations, using 96-well plates and samples appropriately diluted, was established between 7.2 and 2850.6 pg/mL. To perform the ELISA protocol, we used 100 μL of the samples in 1:1 and 1:3 dilutions into each cell, the plates were incubated 90 min at 37 °C. Subsequently, it was washed twice with the washing buffer, 100 μL of a solution of anti-G-CSF antibodies linked with a biotin molecule were added and it was subjected to a new incubation period of 60 min at 37 °C. The plate was washed three times, a further 100 μL of a Streptavidin-HRP containing solution in 1:1000 dilution was added and incubated again at 37 °C for 30 min. Next it was washed five times, 90 μL of the TMB substrate solution in a 1:1000 dilution was added, and it was incubated at 37 °C for other 15 min, which was the time necessary to observe blue color in the samples and in the standard curve. At this time the reaction was stopped with a H_2_SO_4_ solution and the absorbance was measured at 450 nm. The spectrophotometer used was a VersaMaxTunable. Micropate Reader (Molecular Devices, LLC, San José, CA, USA), with a visible absorbance reading range between 340 and 850 nm. Raw data were analyzed using SoftMax Pro Software 5.4 (Molecular Devices, LLC, San José, CA, USA). In all cases, the samples were run in duplicate and internal controls and calibration curve were included in each ELISA Kit. The concentration assigned to samples with an optical density (OD) lower than the limit of detection in the ELISA but higher than the background (zero values) (N = 26) was half of the minimum concentration that could be interpolated in the standard curve. Plasma concentrations of G-CSF were expressed as pg/mL.

### Statistical analysis

Data in Tables 1 and 3 are expressed as the number and percentage of subjects [N (%)] or mean ± standard deviation (mean ± SD). The significance of differences in categorical and normal continuous variables was determined using the chi-square test (or Fisher’s exact test) and Student’s *t*-test, respectively.

**Table 1:** Socio-demographic variables of the sample.

Variables	Group				
	Control (SUD)	SUD		Control (MDD)	MDD
		Cocaine	Alcohol		
Subjects (N)	92	60	65	44	50
Age* years* (Mean ± SD)	40.5 ± 11.8	35.4 ± 7.8	49.4 ± 6.6	44.4 ± 8.9	43.8 ± 8.8
BMI *kg/m*^*2*^ (Mean ± SD)	24.8 ± 3.9	24.3 ± 3.8	26.1 ± 4.7	24.8 ± 4.0	24.8 ± 4.1
**Sex [N (%)]**					
Women	45 (48.9)	18 (30.0)	23 (35.4)	34 (77.3)	40 (80.0)
Men	47 (51.1)	42 (70.0)	42 (64.6)	10 (22.7)	10 (20.0)
**Education [N (%)]**					
Elementary	3 (3.6)	8 (13.3)	27 (41.5)	11 (26.2)	7 (14.0)
Secondary	33 (39.5)	46 (76.7)	23 (35.4)	18 (42.9)	25 (50)
University	48 (57.1)	6 (10.0)	15 (23.1)	13 (31.0)	18 (36)
Employment [N (%)]	73 (89.0)	27 (45.0)	26 (40.0)	32 (76.2)	31 (62.1)

*BMI* body mass index, *MDD* major depressive disorder, *SUD* substance use disorder.

Analyses of covariance (ANCOVA) were performed to evaluate the main effects and interaction of independent variables [e.g., “lifetime SUD diagnosis” (control and SUD; control, cocaine and alcohol), “lifetime MDD diagnosis” (control and MDD) and “sex” (men and women)] on plasma concentrations of G-CSF while controlling for “age” as a covariate. Because plasma concentrations of G-CSF showed a positively skewed distribution and did not pass the D'Agostino-Pearson normality test, raw data were log_10_-transformed to approximate a normal distribution and to ensure statistical assumptions of the ANCOVA. The estimated marginal means and 95% confidence intervals (95% CI) of the log_10_-transformed G-CSF concentrations are represented in the figures, and their back-transformation a into linear scale is included in the description of the results. Post hoc comparisons for multiple comparisons were performed using the Sidak ‘s correction test.

Correlation analyses between plasma concentrations of G-CSF (log_10_-transformed data) and relevant SUD-related variables (i.e., DMS-IV-TR criteria for SUD, duration of abstinence and duration of problematic substance use) were performed using the correlation coefficients of Pearson (r) and Spearman (rho) with continuous and categorical variables, respectively.

The statistical analyses were carried out with GraphPad Prism version 5.04 (GraphPad Software, San Diego, CA, USA) and IBM SPSS Statistics version 22 (IBM, Armonk, NY, USA). A p-value < 0.05 was considered statistically significant.

## Results

### Sociodemographic description of the sample

This study included 311 participants grouped into four experimental groups to explore plasma concentrations of G-CSF: the SUD group, the MDD group and their control groups. Table 1 shows the sociodemographic data for the total sample.

Although the control group for patients with SUD showed comparable age and BMI to the SUD group (mean age of 42.7 years and BMI of 25.2 kg/m^2^), there were differences in the sex composition because the control group was originally sex-balanced in men and women and the SUD group was mainly composed of men (67%). The SUD group was divided into the cocaine subgroup and the alcohol subgroup. In this case, the comparison between both subgroups revealed significant differences in age and education level. Thus, the patients with CUD were younger than the patients with AUD, and the percentage of participants with elementary education was greater in the alcohol subgroup.

A second control group showed sex, age and BMI comparable to those of the MDD group, and no differences were observed across sociodemographic variables. Unlike the SUD group, the MDD group was mostly composed of women (80%).

### Plasma concentrations of G-CSF and age

An initial analysis was performed to explore the correlations between plasma concentrations of G-CSF and variables that could be included in the ANCOVA models as covariates or confounding variables (age and BMI). While log_10_-transformed G-CSF concentrations and BMI were not correlated, log_10_-transformed G-CSF concentrations and age were significantly correlated (r = − 0.233, *p* < 0.001) in the total sample. Interestingly, there were differences in this association based on the group [control group: r = − 0.307, *p* = 0.002; SUD group: r = − 0.165, *p* = 0.047; and MDD group: r =  + 0.070, *p* = 0.707].

### Plasma concentrations of G-CSF and lifetime SUD diagnosis

**Figure 1﻿:**
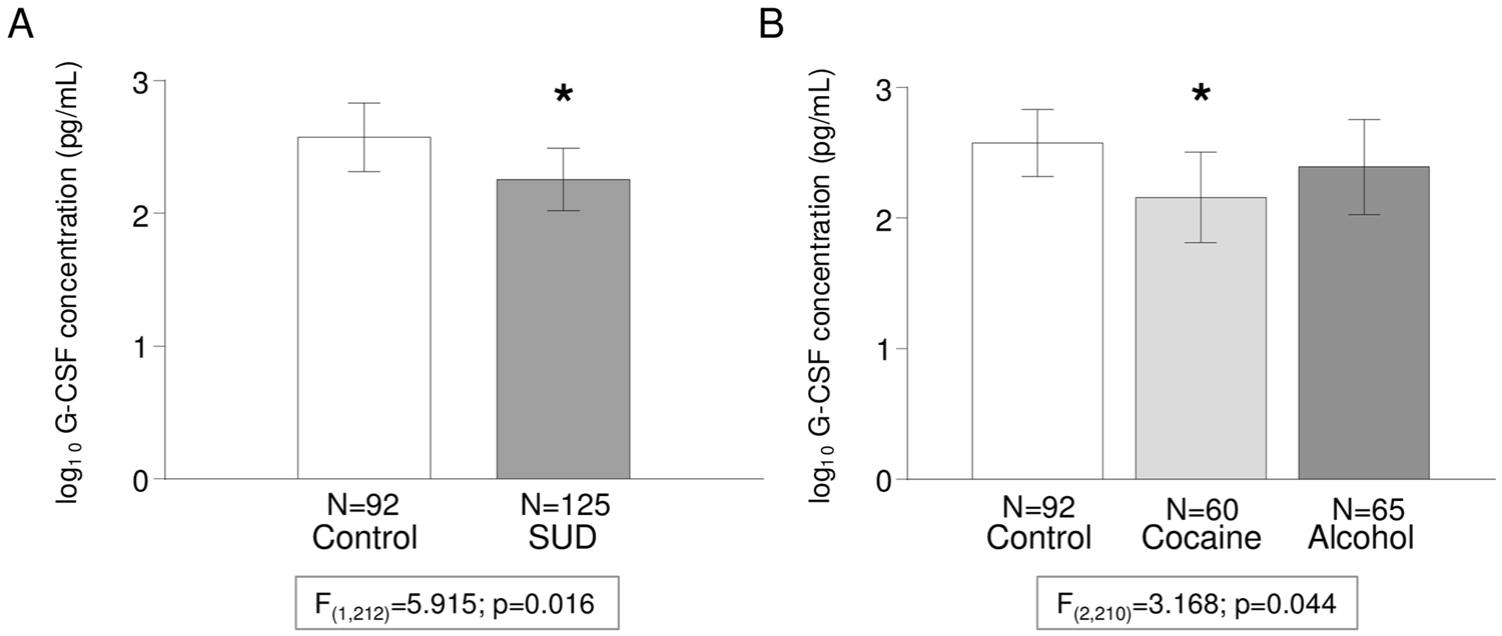
Plasma concentrations of G-CSF and lifetime SUD. (**A**) Bars are estimated marginal means and 95% CI of log_10_-transformed concentrations of G-CSF (pg/mL) in the control and SUD groups based on the “lifetime SUD diagnosis” as factor in ANCOVA. (*) *p* < 0.05 denotes a significant main effect of lifetime SUD; (**B**) Bars are estimated marginal means and 95% CI of log_10_-transformed concentrations of G-CSF (pg/mL) in the SUD subgroups based on the “type of SUD diagnosis” as factor in ANCOVA (*) *p* < 0.05 denotes a significant main effect of type of SUD diagnosis.

The association between lifetime SUD diagnosis and plasma concentrations of G-CSF was first investigated using two-way ANCOVA: raw data for G-CSF concentrations were log_10_-transformed for the analysis with “SUD diagnosis” [SUD (N = 125) and control (N = 92)] and “sex” as factors and “age” as an important covariate. ANCOVA revealed a significant main effect of “SUD diagnosis” on plasma concentrations of G-CSF [*F*_(1, 212)_ = 5.915; *p* = 0.016] (Fig. 1A), and patients with SUD showed significantly lower G-CSF concentrations than control subjects [back-transformation of estimated marginal means: 1414.3 (95% CI 1095.3–1733.2) and 1999.6 (95% CI 1650.2–2349.0) pg/mL, respectively]. In contrast, we found no main effect of “sex” or interaction between the two factors on plasma concentrations of G-CSF.

To elucidate the contribution of each SUD subgroup, we performed a second two-way ANCOVA on log_10_-transformed G-CSF concentrations with “type of SUD diagnosis” [cocaine (N = 60), alcohol (N = 65) and control (N = 92)] as a factor. There was a significant main effect of “type of SUD diagnosis” on plasma concentrations of G-CSF [*F*_(2, 210)_ = 3.168; *p* = 0.044] (Fig. 1B). Specifically, the post hoc test revealed significantly lower G-CSF plasma concentrations in the cocaine subgroup than in the control group [back-transformation of estimated marginal means: 1256.4 (95% CI 768.5–1744.3) pg/mL and 1986.0 (95% CI 1641.8–2330.2) pg/mL, respectively]. Although there was no main effect of “sex”, there was a significant interaction effect between factors [*F*_(2, 210)_ = 3.393; *p* = 0.035] (Figure S1A). The multiple comparison post hoc test showed significant differences in men and women from different groups/subgroups. In male participants, significantly lower G-CSF plasma concentrations were observed in the cocaine subgroup than in the control group (*p* < 0.05) [back-transformation of estimated marginal means: 1110.1 (95% CI 581.8–1638.4) pg/mL and 1977.0 (95% CI 1497.5–2456.5) pg/mL, respectively]. However, significantly lower G-CSF plasma concentrations were observed in the alcohol subgroup than in the control group in women (*p* < 0.05) [back-transformation of estimated marginal means: 1015.7 (95% CI 304.4–1726.9) pg/mL and 1995.0 (95% CI 1501.6–2488.3) pg/mL, respectively].

### Plasma concentrations of G-CSF and relevant SUD-related variables

Because there was a significant association between lifetime SUD diagnosis and plasma concentrations of G-CSF, we explored relevant variables related to cocaine and alcohol addiction: the severity of SUD (based on DSM-IV-TR criteria for AUD and CUD), duration of the last abstinence and problematic substance use. To this end, the SUD group was divided into alcohol and cocaine subgroups, and correlation analyses were performed between plasma concentrations of G-CSF (log_10_-transformed) and these variables (Table 2). The cocaine subgroup had an average of 7.6 DSM-IV-TR criteria for CUD and 55.2 days of abstinence: We found no significant correlations between CUD-related variables and G-CSF concentrations. Regarding the alcohol subgroup, the patients had an average of 7.8 DSM-IV-TR criteria for AUD and 160 days of abstinence: A significant and positive correlation was found between the duration of abstinence and G-CSF concentrations (*p* < 0.05).

**Table 2:** Correlation analyses between plasma concentrations of G-CSF and relevant SUD-related variables in the SUD group.

Variables	Subgroup			
	Cocaine		Alcohol	
	coefficient	*p*-value	coefficient	*p*-value
	G-CSF (pg/mL)^a^		G-CSF (pg/mL)^a^	
DSM-IV-TR criteria for SUD (1–11)	rho = − 0.239	0.066	rho = − 0.092	0.466
Last period of abstinence (*days*) ^a^	r = + 0.117	0.414	r = + 0.295	0.017 *
Problematic substance use (*years*)	r = − 0.043	0.756	r = + 0.106	0.399

^**a**^Log_10_-transformed data.

*Denotes significant difference (p < 0.05).

### Psychiatric comorbidity and medication in patients with SUD

Lifetime SUD is often associated with psychiatric comorbidity (dual diagnosis). We assessed all patients with SUD, and the high prevalence of psychiatric comorbidity was confirmed as a primary characteristic of the sample: 64 patients with SUD (51.6%), including 19 patients with CUD (31.7%) and 45 patients with AUD (69.2%), were diagnosed with other mental disorders. The most prevalent mental disorders in the SUD group were as follows: 31.7%, mood disorders; 27%, anxiety disorders; 15%, cluster B personality disorders; and 4.8%, psychotic disorders. No significant differences were found in the sex proportions among these comorbid mental disorders.

Table 3 shows main mental disorders diagnosed based on DSM-IV-TR criteria in the cocaine and alcohol subgroups.

**Table 3:** Psychopathological characteristics of the cocaine and alcohol subgroups.

Variables	Subgroup		
	Cocaine	Alcohol	*p-*value ^a^
Major depressive disorder (MDD) [N (%)]	11 (18.3)	29 (44.6)	0.002 *
Dysthymia disorder [N (%)]	3 (5.0)	2 (3.1)	0.672
Cyclothymic disorder [N (%)]	1 (1.7)	1 (1.5)	1.000
Schizophreniform disorder [N (%)]	1 (1.7)	–	–
Psychotic disorder not specified [N (%)]	1 (1.7)	5 (7.7)	0.210
Social phobia [N (%)]	3 (5.0)	2 (3.1)	0.670
Panic disorder [N (%)]	2 (3.3)	10 (15.4)	0.032 *
Agoraphobia disorder [N (%)]	2 (3.3)	1 (1.5)	0.670
Generalized anxiety disorders [N (%)]	2 (3.3)	3 (4.6)	1.000
Obsessive–compulsive disorder [N (%)]	6 (10.0)	1 (1.5)	0.054
Post-traumatic stress disorder (PTSD) [N (%)]	6 (10.0)	7 (10.8)	1.000
Anorexia disorder [N (%)]	2 (3.3)	1 (1.5)	0.607
Bulimia disorder [N (%)]	3 (5.0)	3 (4.6)	1.000
Antisocial personality disorder [N (%)]	2 (3.3)	3 (4.6)	1.000
Borderline personality disorder [N (%)]	5 (8.3)	10 (15.4)	0.277
Attention deficit hyperactivity disorder (ADHD) [N (%)]	10 (16.7)	6 (10.2)	0.421

^**a**^Data were analyzed using chi-square test or Fisher’s exact test.

*Denotes significant difference (p < 0.05).

As expected, the elevated prevalence of psychiatric comorbidities was associated with high use of psychoactive medications. Notably, 66.7% of patients with SUD were using medication during the last 12 months: antidepressants (40.7%), anxiolytics (40.7%), anticonvulsants (22.8%), antipsychotics (8.9%) and *disulfiram* (39%). To investigate the potential effects of psychiatric medication on G-CSF concentrations, we performed two-way ANCOVA with “psychiatric medication” (nonmedicated and medicated patients with SUD) and “sex” as factors and “age” as a covariate. However, the analysis revealed no main effects of “psychiatric medication” on log_10_-transformed G-CSF concentrations (Table 4). In addition, no significant main effect of “sex” on G-CSF concentrations or interaction between factors was observed.

**Table 4:** Plasma concentrations of G-CSF and use of psychiatric medication and disulfiram during the last year in the SUD group.

Variables	SUD group		ANCOVA statistics ^a^		
	Non-medicated	Medicated	*F*	df	*p*-value
	Log_10_ G-CSF (pg/mL) [Mean (95%CI)]				
Antidepressants	2.269 (1.970–2.568)	2.299 (1.934–2.665)	0.015	1,119	0.901
Anxiolytics	2.283 (1.985–2.582)	2.278 (1.913–2.643)	0.000	1,119	0.983
Anticonvulsants	2.238 (1.983–2.494)	2.427 (1.950–2.904)	0.471	1,119	0.494
Antipsychotics	2.230 (1.998–2.463)	2.798 (2.055–3.541)	2.081	1,119	0.152
*Disulfiram*	2.330 (2.038–2.622)	2.205 (1.837–2.574)	0.262	1,119	0.610

^**a**^Data were analyzed using ANCOVA (medication during the last year, sex, age).

### Plasma concentrations of G-CSF and comorbid MDD in patients with SUD

Because we observed no significant effect of “psychiatric medication”, we analyzed G-CSF concentrations in the SUD group based on the most prevalent comorbid mental disorders using two-way ANCOVA with “sex” as a factor while controlling for “age” but not for “psychiatric medication”. The analyses revealed a significant association between log_10_-transformed G-CSF concentrations and “comorbid MDD diagnosis” but not with other comorbid mental disorders (data not shown).

**Figure 2:**
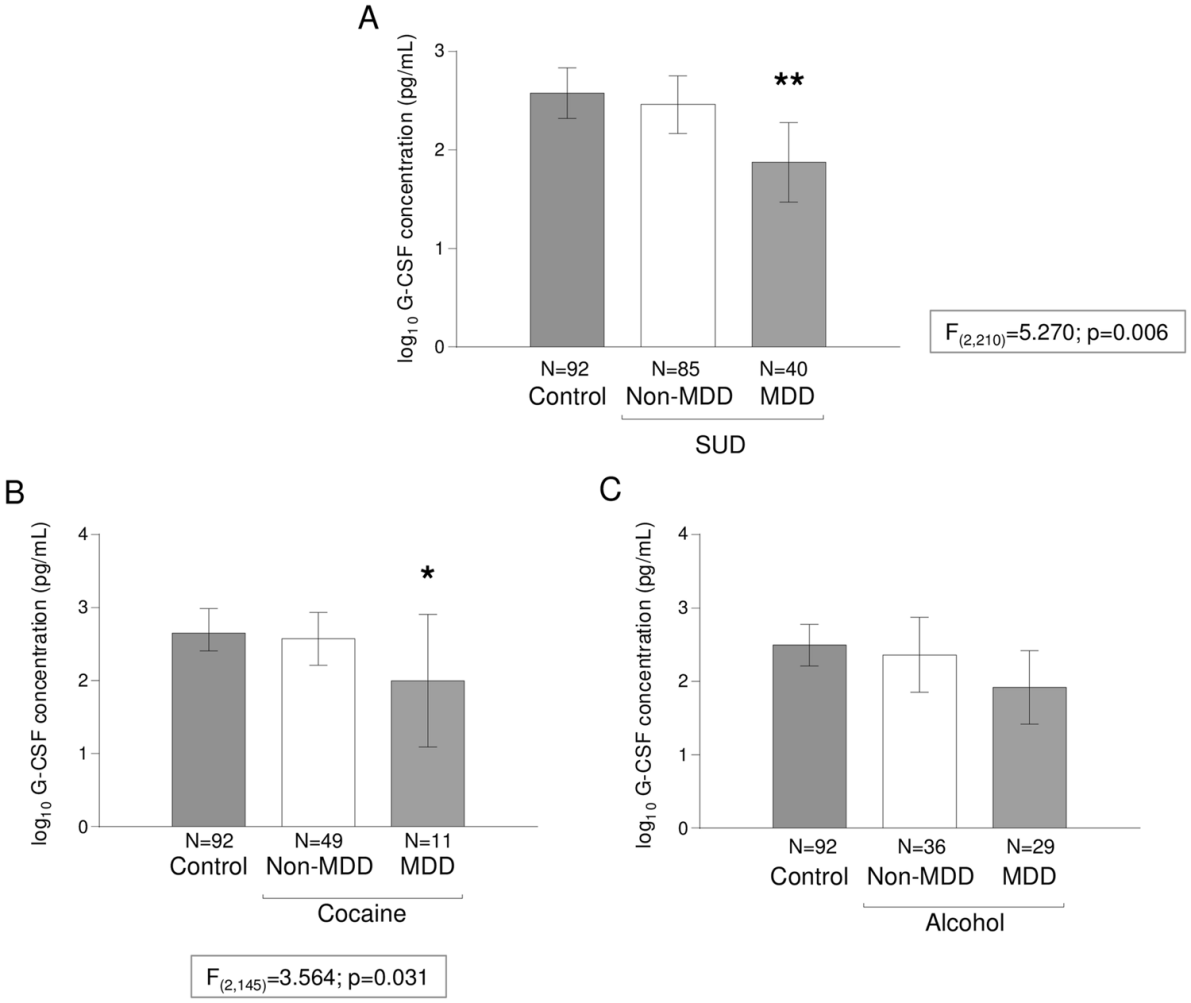
Plasma concentrations of G-CSF and comorbid MDD in patients with SUD. (**A**) Bars are estimated marginal means and 95% CI of log_10_-transformed concentrations of G-CSF (pg/mL) in the control and SUD groups based on the “comorbid MDD diagnosis” as factor in ANCOVA. (**) *p* < 0.01 denotes a significant main effect of comorbid MDD diagnosis in patients with SUD; (**B**) Bars are estimated marginal means and 95% CI of log_10_-transformed concentrations of G-CSF (pg/mL) in the cocaine subgroup based on the “comorbid MDD diagnosis” as factor in ANCOVA. (*) *p* < 0.05 denotes a significant main effect of comorbid MDD diagnosis; (**C**) Bars are estimated marginal means and 95% CI of log_10_-transformed concentrations of G-CSF (pg/mL) in the alcohol subgroup based on the “comorbid MDD diagnosis” as factor in ANCOVA.

Therefore, there was a significant main effect of “comorbid MDD diagnosis” on plasma concentrations of G-CSF [*F*_(2, 210)_ = 5.270; *p* = 0.006] (Fig. 2A). The post hoc test revealed that patients with SUD and comorbid MDD had significantly lower G-CSF concentrations than control subjects (*p* < 0.01) [back-transformation of estimated marginal means: 934.8 (95% CI 388.7–1480.8) pg/mL and 2007.5 (95% CI 1661.5–2353.5) pg/mL, respectively]. Regarding the “sex” factor, there was no main effect or interaction on plasma concentrations of G-CSF.

#### Plasma concentrations of G-CSF and type of comorbid MDD

Because comorbid MDD can be classified according to DSM-IV-TR criteria into primary and substance-induced MDD, we explored whether the low plasma concentrations of G-CSF in patients with SUD and comorbid MDD were associated with a specific type of MDD. However, the statistical analysis revealed no main effect of “type of comorbid MDD” on log_10_-transformed G-CSF concentrations (Table 5) and, therefore, no differences among patients with SUD and primary MDD and/or substance-induced MDD.

**Table 5:** Plasma concentrations of G-CSF and type of comorbid MDD in the SUD group.

Variables	SUD group				ANCOVA statistics ^a^		
	Non-MDD	Primary MDD	Substance-induced MDD	Both MDD	*F*	df	*p-*value
Subjects (N)	85	15	16	9			
Log_10_ G-CSF (pg/mL) [Mean (95%CI)]	2.481 (2.213–2.750)	1.789 (1.139–2.439)	2.169 (1.544–2.793)	1.687 (0.817–2.557)	2.938	3,119	0.128

^**a**^Data were analyzed using ANCOVA (type of MDD, sex, age).

#### Plasma concentrations of G-CSF and comorbid MDD in the cocaine and alcohol subgroups

Then, we investigated the association between G-CSF concentrations and comorbid MDD diagnosis in the SUD group while separately considering cocaine and alcohol subgroups. For each subgroup, two-way ANCOVA was used with “comorbid MDD diagnosis” and “sex” as factors while controlling for “age”.

The analysis in the cocaine subgroup showed a significant main effect of “comorbid MDD diagnosis” on plasma concentrations of G-CSF [*F*_(2, 145)_ = 3.564; *p* = 0.031] (Fig. 2B). However, the post hoc test revealed no significant differences between patients with CUD. In addition, there was no main effect of “sex” or interaction between factors on plasma concentrations of G-CSF.

Regarding the alcohol subgroup, there was no main effect of “comorbid MDD diagnosis” on plasma concentrations of G-CSF (Fig. 2C). In contrast, there was a significant main effect of “sex” [*F*_(1, 150)_ = 4.384; *p* = 0.038] (Figure S1B), and women had significantly lower G-CSF concentrations than men [back-transformation of estimated marginal means: 1361.9 (95% CI 855.2–1868.6) pg/mL and 2040.0 (95% CI 1640.6–2439.4) pg/mL, respectively]. There was no interaction between “comorbid MDD diagnosis” and “sex”.

### Plasma concentrations of G-CSF and MDD diagnosis

**Figure 3:**
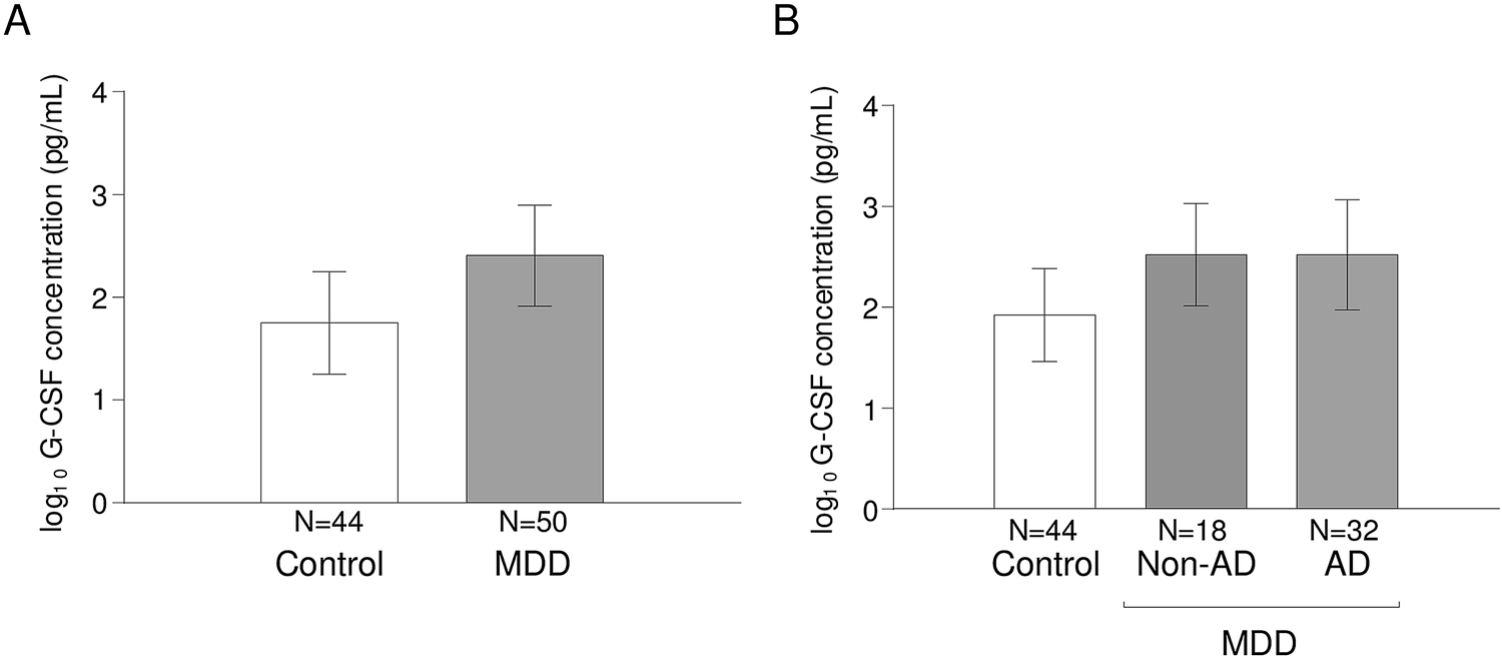
Plasma concentrations of G-CSF and MDD diagnosis. (**A**) Bars are estimated marginal means and 95% CI of log_10_-transformed concentrations of G-CSF (pg/mL) in the control and MDD groups based on the “MDD diagnosis” as factor in ANCOVA; (**B**) Bars are estimated marginal means and 95% CI of log_10_-transformed concentrations of G-CSF (pg/mL) in the MDD subgroups based on the “antidepressant medication use” as factor in ANCOVA.

Given that there was a significant association between G-CSF concentrations and comorbid MDD diagnosis in the SUD group, we examined plasma concentrations of G-CSF in patients from primary-care settings who were diagnosed with primary MDD but no lifetime SUD. Similar to the previous analysis, two-way ANCOVA was performed with “MDD diagnosis” and “sex” as factors while controlling for “age”. However, there were no main effects of “MDD diagnosis”, “sex” or interaction on log_10_-transformed G-CSF concentrations (Fig. 3A).

Finally, because many patients with MDD were using psychiatric medications during the study as part of their treatment [primarily antidepressants (36%) and anxiolytics (40%)] and recent research in our group has reported high plasma concentrations of antiinflammatory molecules (e.g., *N*-acylethanolamines) in patients with MDD and antidepressant treatment^48^, we investigated the effects of antidepressants and anxiolytics on G-CSF concentrations. However, the analyses revealed no significant main effects of “antidepressants” on log_10_-transformed G-CSF concentrations (Fig. 3B). There was also no main effect of “anxiolytics”.

## Discussion

In the present exploratory study, we examined G-CSF concentrations in the plasma of abstinent patients with lifetime SUD from outpatient treatment programs, patients with MDD from primary-care settings and control healthy subjects. In addition, all participants were characterized through different psychiatric/clinical assessments based on the DSM-IV-TR criteria. The main findings are summarized as follows: (1) Plasma concentrations of G-CSF were significantly and inversely correlated with age in the control group and the SUD group, but the correlation was nonsignificant in the MDD group; (2) G-CSF concentrations were significantly lower in the patients with SUD than in the control subjects, specifically in the cocaine subgroup; (3) The patients with SUD and comorbid MDD had significantly lower G-CSF concentrations than the patients with SUD but not comorbid MDD or control subjects; (4) G-CSF concentrations were significantly lower in the cocaine subgroup with comorbid MDD but not in the alcohol subgroup with comorbid MDD; and (5) The patients with MDD showed no differences in plasma concentrations of G-CSF compared with their control subjects. The results suggest a potential role of this immunomodulatory factor in MDD disorders associated with SUDs rather than in primary MDD disorders.

Consistent with our findings, it is known that G-CSF is close and negatively correlated with age as a typical growth factor. Previous studies have reported G-CSF as a risk factor for cognitive impairment in preclinical models and as one of the 18 plasma signaling proteins that are collectively predictive of conversion from mild cognitive impairment to Alzheimer’s disease^49,50^. Unlike the control group, our data showed fluctuations in the inverse association between G-CSF concentrations and age across the different groups of patients. Thus, the inverse association was attenuated in the patients with SUD and nonsignificant in the patients with MDD. This suggests that a history of MDD could be associated with disturbances in the immune system through immunomodulatory growth factors.

However, our results confirm that changes in the plasma concentrations of G-CSF might be attributed to the history of addiction in patients with AUD and CUD, although the nature of this relationship demands further research. While in preclinical studies this immunomodulatory growth factor helps to consolidate cocaine reward/cocaine seeking behavior, in abstinent humans we observed that G-CSF concentrations are lowered in patients with CUD and with a nonsignificant trend toward a negative correlation with the severity of CUD. Because there is a lack of data on the acute effects of cocaine on circulating concentrations of G-CSF, we can only speculate on the nature of this finding. In accordance with the allostatic model set in place for most biological modulatory transmitters related to addiction^51^, whether G-CSF treatment in preclinical models of cocaine reward is associated with boosting or sustaining cocaine-seeking behavior^40^, it is reasonable to consider that G-CSF might be downregulated during a period of abstinence after chronic cocaine use in severe cocaine-addicted patients. However, these results regarding cocaine need to be conclusively determined. Regarding alcohol, G-CSF has been linked to alcohol-related liver disease, and a study is currently validating the efficacy of liver regeneration through G-CSF therapy^52^. Unfortunately, we have no data regarding accessory digestive organ diseases in the present sample to test this association. However, previous studies in patients with AUD from the same source as our group reported a high prevalence of alcohol-related liver and pancreas diseases^4,53^. In contrast, our study revealed a positive correlation between plasma concentrations of G-CSF and the duration of the last abstinence from alcohol. Similar to G-CSF, other inflammation-related signals in the plasma [such as the lipid messenger *N*-oleoilethanolamine (OEA)] were found to be related to the duration of alcohol abstinence. Thus, OEA decreases as the duration of alcohol abstinence increases in patients with AUD, and it has been proposed as a marker of abstinence^54^.

Our results also showed that plasma concentrations of G-CSF were lower in the patients with SUD and comorbid MDD but not in the patients with primary MDD without SUD. Long-lasting stress effects have been studied in the development of mood disorders^55^, with several forms of dysregulation observed in relation to proinflammatory status^56^. Recent studies from our group have described differences in other peripheral inflammatory mediators relative to the presence of comorbid psychiatric disorders in patients with AUD. In particular, decreased concentrations of the chemokine eotaxin-1 (CCL11) in the plasma of abstinent patients with AUD have been associated with comorbid mood and anxiety disorders^14^. Another study reported lower concentrations of IL-1β, CXCL12 and CCL11 in cocaine-induced MDD^57^. Although we observed lower concentrations of G-CSF in patients with CUD and comorbid MDD, these changes in the concentrations were not linked to the type of MDD (primary MDD, substance-induced MDD and both). How the interaction of SUD and MDD affects G-CSF must be elucidated with further research.

Furthermore, this immunomodulatory growth factor has also been studied in relation to memory functions in a preclinical model, showing that deficiencies in G-CSF concentrations in the hippocampus decreased spatial learning performance and memory formation^58^. Therefore, we consider that it would be interesting to measure the role of G-CSF in a neuropsychological cohort of patients with SUD and substance-induced memory deficits.

## Conclusions and limitations

These findings support the importance of monitoring G-CSF in the context of SUD and psychiatric comorbidity, but we are aware of the limitations of this exploratory study: (1) The high prevalence of psychiatric comorbidity in patients with SUD increased the difficulty in the characterization of a specific comorbid disorder such as MDD; (2) There are additional uncontrolled biological and sociodemographic variables that were not included in the present study but could be a source of variability in G-CSF concentrations; (3) The findings were restricted based on statistical limitations such as compliance of parametric assumptions and/or appropriate transformations of raw data (e.g., using log_10_-transformation), calculation of sample size, number of independent variables/covariates in the linear models, etc. Finally, it is important to replicate these findings with larger samples in cohorts from different geographical and cultural backgrounds, as well as to characterize G-CSF concentrations in longitudinal studies during critical stages in the course of MDD and SUD, such as substance abstinence and active substance use.

In conclusion, these findings support an association between lifetime SUD and plasma concentrations of G-CSF, with lowered concentrations in abstinent patients with CUD. Interestingly, we found lower plasma concentrations of G-CSF in association with comorbid MDD diagnosis in these cocaine-addicted patients. Additionally, plasma concentrations of G-CSF correlated with alcohol-related variables such as the duration of abstinence, an interesting variable related to a good prognosis for those with SUD. Moreover, a dysregulation in G-CSF concentrations was observed with comorbid MDD in patients with lifetime SUD but not with primary MDD without SUD. This association with comorbid MDD was independent of the type of MDD (comorbid substance-induced MDD or comorbid primary MDD). Further research is necessary to elucidate the role of G-CSF as a potential biological marker of immunomodulatory and inflammatory states in addiction, mood disorders and dual diagnosis.

## Supplementary Information

Supplementary Information.
