# Supplementary material for: Plasma concentrations of granulocyte colony-stimulating factor (G-CSF) in patients with substance use disorders and comorbid major depressive disorder
Source: Sci Rep. 2021 Jul 1;11:13629. doi: 10.1038/s41598-021-93075-1 (PMC8249412; doi:10.1038/s41598-021-93075-1)
Supplement: Supplementary file 1 — Supplementary Information. [file 41598_2021_93075_MOESM1_ESM.docx]

**Supplementary material**

**Plasma Concentrations of Granulocyte Colony-Stimulating Factor (G-CSF) in Patients with Substance Use Disorders and Comorbid Major Depressive Disorder**

Sandra Torres Galván^1,2,3#^; María Flores-López^1,2,4#^; Pablo Romero-Sanchiz^1,2,5^; Nerea Requena-Ocaña^1,2^; Oscar Porras-Perales^1,2,4,6^; Raquel Nogueira-Arjona^1,2,5^; Fermín Mayoral^1,2^; Pedro Araos^1,2,4^; Antonia Serrano^1,2^; Roberto Muga^7,8^; Francisco Javier Pavón^1,2,6,9^*; Nuria García-Marchena^1,2,7^*; Fernando Rodríguez de Fonseca^1,2^*

^1^ Unidad de Gestión Clínica de Salud Mental, Hospital Regional Universitario de Málaga, Málaga, Spain

^2^ Instituto de Investigación Biomédica de Málaga-IBIMA, Málaga, Spain

^3^ Facultad de Farmacia, Universidad Complutense de Madrid, Madrid, Spain

^4^ Facultad de Psicología, Universidad de Málaga, Málaga, Spain

^5^ Department of Psychology, University of Roehampton, London, UK

^6^ Unidad de Gestión Clínica del Corazón, Hospital Universitario Virgen de la Victoria, Málaga, Spain

^7^ Unidad de Adicciones, Servicio de Medicina Interna, Institut D’Investigació en Ciències de la Salut Germans Trias i Pujol (IGTP), Badalona, Spain

^8^ Departamento de Medicina, Universitat Autònoma de Barcelona, Barcelona, Spain

^9^ Centro de Investigación Biomédica en Red Enfermedades Cardiovasculares (CIBERCV), Instituto de Salud Carlos III, Madrid, Spain

**^#^** These authors contributed equally to this study

***** Correspondence

Francisco Javier Pavón ([javier.pavon@ibima.eu](mailto:javier.pavon@ibima.eu)). Laboratorio de Medicina Regenerativa, Hospital Regional Universitario de Málaga, Avda. Carlos Haya 82, sótano. Málaga, 29010, Spain

Nuria García-Marchena (ngarciam@igtp.cat). Unidad de Adicciones- Servicio de Medicina Interna. Institut D’Investigació en Ciències de la Salut Germans Trias i Pujol (IGTP). Campus Can Ruti, Carrer del Canyet s/n, Badalona, 08916, Spain

Fernando Rodríguez de Fonseca (fernando.rodriguez@ibima.eu). Laboratorio de Medicina Regenerativa, Hospital Regional Universitario de Málaga, Avda. Carlos Haya 82, sótano. Málaga, 29010, Spain

**
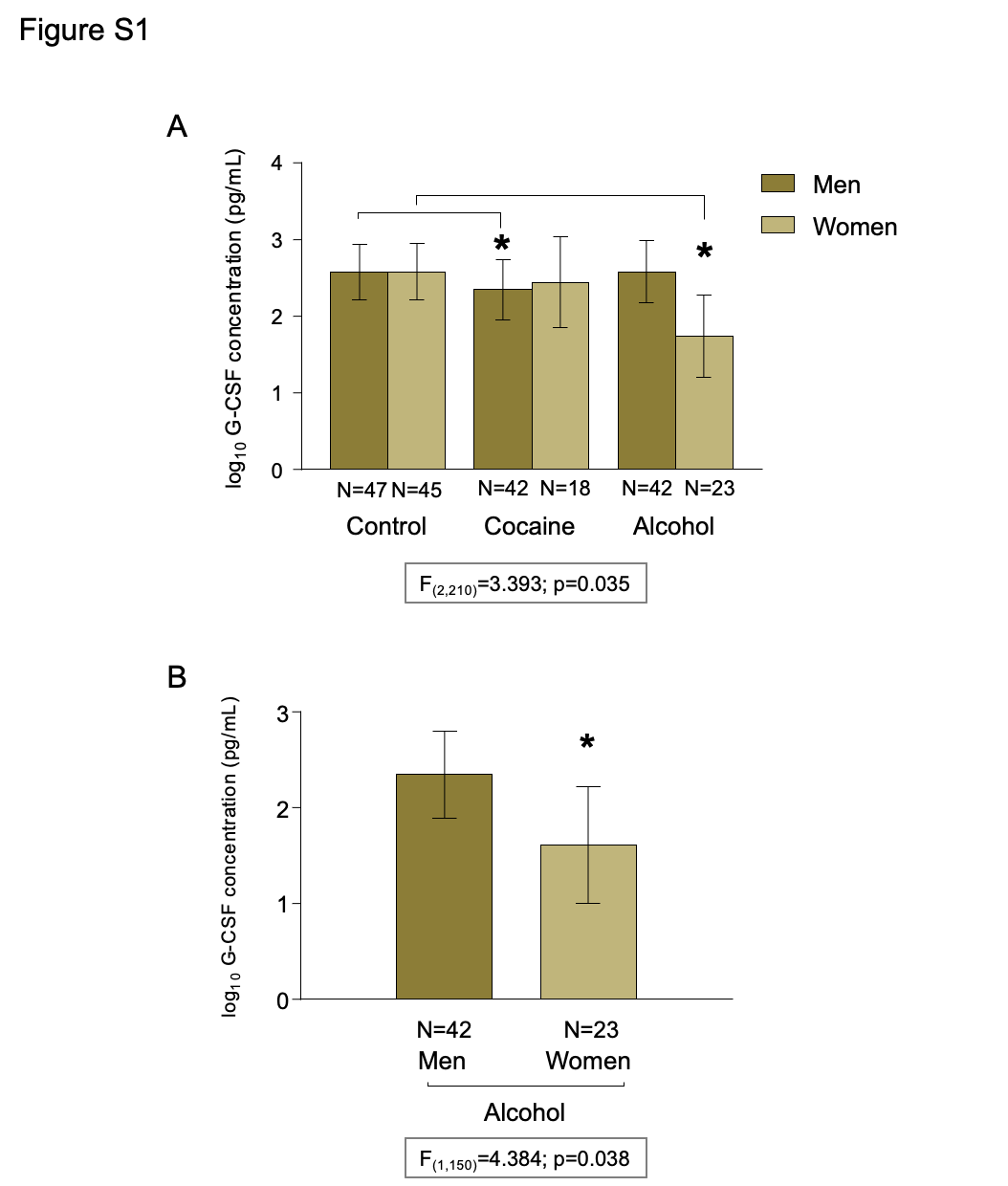
**

**FIGURE S1. Plasma concentration of G-CSF and sex in patients with SUD. (A)** Bars are estimated marginal means and 95% CI of log_10_-transformed concentrations of G-CSF (pg/mL) in the SUD subgroups based on the interaction effect between “sex” and “lifetime SUD diagnosis” as factors in ANCOVA. (*) *p*<0.05 denotes significant differences compared with men and women from the control group using multiple comparisons; **(B)** Bars are estimated marginal means and 95% CI of log_10_-transformed concentrations of G-CSF (pg/mL) in the alcohol subgroup based on “sex” as factor in ANCOVA. (*) *p*<0.05 denotes a significant main effect of sex.
